# Shockwave Therapy Versus Dry Needling for the Management of Iliotibial Band Syndrome: A Randomized Clinical Trial: SWT Vs. DN for the Treatment of ITBS

**DOI:** 10.31661/gmj.v10i0.2174

**Published:** 2021-07-06

**Authors:** Maghroori Razie, Karshenas Leila, Khosrawi Saied

**Affiliations:** ^1^ Department of Physical Medicine and Rehabilitation, School of Medicine, Isfahan University of Medical Sciences, Isfahan, Iran

**Keywords:** Iliotibial Band Syndrome, Dry Needling, Extracorporeal Shockwave Therapy, Pain

## Abstract

Background: Iliotibial band syndrome (ITBS) is a common leading cause of lateral knee pain. Despite varieties of medical and non-medical treatments proposed for the management of ITBS, the best therapeutic approach for its treatment remained a significant question. The current study aims to compare the outcomes of dry needling (DN) versus shockwave therapy (SWT) in the management of ITBS. Materials and Methods: This randomized clinical trial was conducted on 40 patients diagnosed with ITBS. The patients were randomly divided into two treatment groups of DN (n=20) and SWT (n=20). Visual analog scale for the pain assessment, Lower Extremity Functional Scale (LEFS) for the function evaluation, and length of the iliotibial band were assessed at baseline, immediately after the cessation of the intervention, and within four weeks. Results: The two groups were similar regarding demographic characteristics (P0.05). Both approaches could efficiently lead to improved pain (P0.001) and promoted function based on LEFS (P0.001); however, iliotibial band length (ITBL) did not alter remarkably (P0.05). The groups were similar in terms of pain score, LEFS, and ITBL at all of the assessment intervals (P0.05), but the pain score within four weeks following the interventions that were significantly better in DN (P=0.023). Conclusion: Based on our results, DN, as well as SWT, could remarkably lead to an improvement in pain and function among patients resenting from ITBS; however, none of the approaches was superior over the other.

## Introduction

IIliotibial band syndrome (ITBS) is a leading cause of pain complaints in the lateral aspect of the knee and is mostly notified in runners [1]. ITBS is an overuse syndrome probably occurring because of friction between the iliotibial band (ITB) and the lateral epicondyle of the knee, while the knee is about 30 degrees flexed.This continual process poses an inflammation in the distal part of the ITB, leading to notifying severe disabling pain in the knee, lateral thigh, and hip and prevents a person from participating in physical activities [2,3].Length of the leg and increase in the lateral femoral epicondyle prominence are the non-modifiable factors associated with ITBS and the modifiable factors such as muscle weakness in the hip abductor muscles, particularly reduced flexibility of the hip, and abnormal kinematics of the lower extremity [1]. Despite varieties of theories proposed for the underlying etiology of ITBS, the therapeutic approach for its treatment remained a major question. The patients with mild symptoms are primarily treated medically using non-steroidal anti-inflammatory drugs (NSAIDs); however, numerous biopsies opposed the presence of inflammation [1]. Physical therapies, static stretching, strengthening, manual therapy, deep friction massage, and appropriate shoe worn, plus training schedules, are the other recommended options for ITBS treatment. In cases with long-term irresponsive ITBS, surgery has been used as the last alternative [3,4,5]. Nevertheless, the surgical procedure is invasive, and the outcomes of the other approaches were controversial [1]. Dry needling (DN), as well as shockwave therapy (SWT), has been successfully used for various musculoskeletal disorders such as myofascial pain, enthesitis, tendonitis, fasciitis, and trigger point [6,7,8,9]. However, the information about the efficacy of DN for ITBS is limited to some case reports and only a cease series [10,11,12], and a few studies have assessed SWT [13]. Therefore, the current study was designed to investigate the efficacy of DN versus SWT in ITBS management.

## Materials and Methods

1. Study Population This randomized controlled clinical trial was conducted on 40 patients diagnosed with ITBS and referred to the Physical medicine and Rehabilitation outpatient clinics affiliated with Isfahan University of Medical Sciences from May 2018 to August 2019. The study protocol was approved by the Ethical Committee of Isfahan University of Medical Sciences (code number: IR.MUI.MED.REC.1398.161).Besides, this report as a phase 3 trial was proposed for the Iranian Registry of Clini¬cal Trials and legislated based on the code IRCT20190824044598N1. The protocol was explained to the patients, they were reassured about the confidentiality of their personal information, and written consent was obtained. Patients (ranged 18-60 years old) with documented ITBS diagnoses who had normal neurological examinations were included. ITBS was diagnosed by an expert physical medicine and rehabilitation specialist according to the clinical physical examination, positive Ober's test, and the presence of the least one trigger point on an ITB. Any fracture in thigh, knee, or shin or any surgical procedure on the affected knee within the last 12 months, application of any thera¬peutic intervention to control the chronic knee pain (i.e., physiotherapy and/or injections at trigger points) within two months, and the use of NSAIDs for over two weeks before the interventions, radiculopathies, coagulopathy, and anticoagulant agents use were considered the unmet criteria. Reluctance to participate in the study, over 20% defects in the medical records, and failure to participate in reassess¬ments were the exclusion criteria of the current study. Convenience sampling was administered to include the studied population. After that, the participants were randomly allocated to DN therapy and SWT using Random Allocation Software (Excel software, Microsoft Office 2010, The United States). Therefore, the patients with odd numbers were allocated to DN therapy and those with even numbers to the shockwave therapy. The person who gathered the data about the outcomes of the interventions was blinded to the type of treat¬ment. 2. Interventions The trigger points were primarily found based on pincer palpation on the lateral aspect of the thigh and lateral femoral epicondyle. To keep the location of the trigger points between the sessions of interventions, a 10*10 cm centrally perforated piece of a paper was administered [14]. 2.1. DN Therapy The patients allocated to the DN group under¬went the intervention twice a week for four weeks. A skilled target physical medicine and rehabilitation specialist performed this technique in a sterile condition to minimize the potential bias. The patient was asked to sleep on the opposite side of the affected leg; the painful leg was upward and put a pillow between the legs. The interventionist cleaned the penetration site with isopropyl alcohol 70%, then wore sterile gloves and performed the needling. In this term, the trigger point firmly held between the thumb and the point¬ing finger by non-dominant hand and a 0.25 mm, 25-gauge needle by the dominant hand. Therefore, the needle was rapidly inserted into the trigger point via a 30° angle and taken out at low speed. Following the inser¬tion of the needle, a local twitch response may occur. Therefore, the fanning technique was applied in which the needle was repeatedly inserted into diverse parts of the trigger point and pulled out as long as there was no further twitch. Eventually, the needle was preserved for 15 minutes to achieve the analgesic effects [15, 16]. 2.2. SWT The latter group of the patients underwent SWT once a week for four weeks. The pa¬tients slept laterally with 30° angle between the thighs and shins. No local anesthesia was administered. The SWT was done by electromagnetic type Dornier AR2 machine (Storz Medical, Tager¬wilen, Switzerland) radial probe. SWT was initiated using 500 pulses at 0.10 mJ/mm2 (2Bar) with 15 Hz frequency to the lateral femoral condyle to adjust to the treatment. An additional 2000 pulses were applied at 0.10mJ/mm2–0.4 mJ/mm2 (2–4 Bar), 15 Hz, depending on pain tolerance. Eventually, three lateral thigh trigger points were treated [13]. All of the patients (regardless of the in¬tervention) were trained to perform a similar stretching ITB exercise. The performance of exercises was recalled and checked through telephoning by the study's correspondents twice a week. In cases with pain compliant, only every 6-hour oral acetaminophen 500 mg was allowed. 3. Means of Assessment All of the patients were followed for four post-intervention weeks, and the evaluations were performed at baseline, immediately by the last session of interventions, and within four weeks after the intervention cessation. In order to minimize the probable bias, all the as¬sessments were performed by a skilled physi¬cal medicine and rehabilitation specialist. The demographic characteristics, including age and gender, were recorded in the study check¬list. A visual analog scale (VAS) was applied to assess pain severity. Lower Extremity Func¬tional Scale (LEFS) and iliotibial band length (ITBL) then further evaluated parameters. 3.1. VAS The VAS score is a self-reported scale to assess the pain severity ranging from 0 to 10, representing the least to the most severe pain sensation [17]. 3.2. LEFS LEFS is a questionnaire containing 20 items assessing the lower extremity function based on the intensity of the related activities' performance. This scoring system is designed based on the five-score Likert scale ranging from zero as the worst condition to four as without bothersome. This scale scores from zero to eighty, and the higher score represent a better condition. Negahban et al. have vali¬dated the Persian version of LEFT with Cron¬bach's alpha of above 0.70 for each item [18]. 3.3. The length of the iliotibial band Ober test was administered to assess the normality of ITBL as well as its measurement. The patient was asked to sleep on the opposite side of the affected leg; therefore, the painful leg was upward. The lower leg was flexed in hip and knee joints. The examiner put one of the hands on the hip joint and the other under the examined knee. Then, the affected knee was 90° flexed without any rotation in the hip joint, where the hip joint was extended simul¬taneously. At this moment, the examiner takes the hand, which holds the knee away. If the ITBL was normal, the gravity pulls the knee down at a level under the bed, and the test was interpreted as negative; while in the shortened bands, the thigh was stopped at levels higher than the bed. The distance between the bed to the medial aspect of the patella was measured using a calibrator ruler [19]. 4. Statistical Analysis The obtained data were entered into the Statistical Package for Social Sciences (SPSS, version 25, IBM Corporation, Armonk, NY, USA). Continuous data were presented in mean and standard deviation (SD). Absolute numbers and percentages were administered to present qualitative information. Fisher's exact test, Mann-Whitney test, t-test, repeated measure ANOVA, and generalized estimating equation (GEE) test were utilized for analyt¬ics. A P=0.05 was determined as the level of significance.

## Results

**Figure 1:**
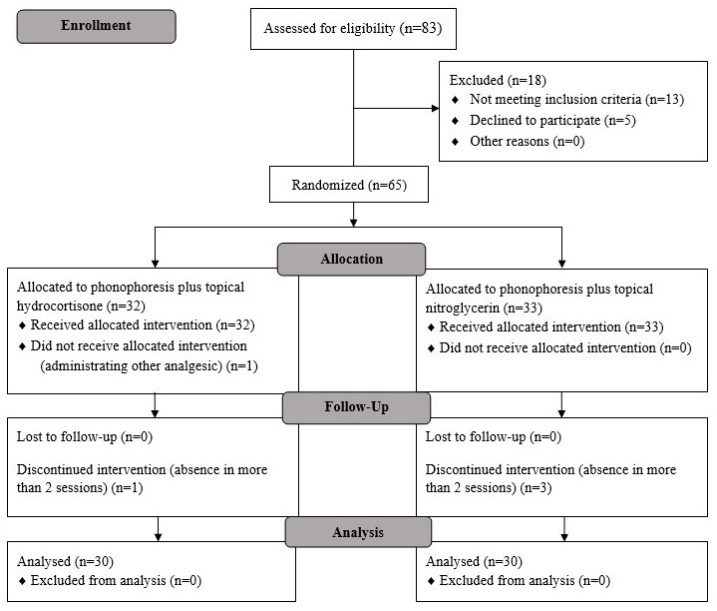
Consort diagram of the studied population.

In the current study, the eligibility of 48 cases with the diagnosis of ITBS has been evaluated; among them 40 patients met the criteria for participation in the study and were randomly allocated to the DN therapy (n=20) and SWT (n=20, Figure-1). The mean age, body mass index (BMI), and the duration of ITBS of the studied popula¬tion was 51.83±14.64 (range:22-78 years), 25.98±3.64 (range:19.10-34.55 kg/m2), and 28.4±17.25 (0.5-120 months), respectively. The comparison of the two groups regarding the demographic data revealed no significant differences (P>0.05, Table-1). The baseline pain assessment showed no significant difference between the two groups (P=0.38) as well as the comparison imme¬diately following the intervention cessation (P=0.39); however, the pain score was remark¬ably less in the DN-treated group (P=0.023). Both of the interventions led to a significant improvement of pain by the time in each group (P<0.001). Besides, in contrast to lacking any differ¬ence between the two groups at the interval assessments of LEFS in any of the evaluations (P>0.05), repeated measure ANOVA showed a significant change in the LEFS scores of both the treatment approaches (P<0.001, Table-2). None of the interventions showed a statistically remarkable alteration in the ITBL (P>0.05). In addition, the comparison of ITBL at base¬line between the groups showed no difference (P=0.56) as well as the assessments at the time of intervention cessation (P=0.86) and within four weeks post-intervention (P=0.79). None of the administered approaches was accompa¬nied by any significant complication.

## Discussion

The current study was conducted to compare the efficacy of SWT versus DN to manage cases with ITBS. The two assessed groups were similar in terms of demographic charac¬teristics; therefore, the possible confounding role of these factors is eliminated, and the results are dedicatedly contributed to the treat¬ment approach. Both treatments could properly improve the pain and LEFS, but not ITBL. The comparison of DN versus SWT generally revealed that the approaches were non-inferior over the other; however, those under the DN approach experienced significantly less severe pain in the four-week follow-up investigation. Limited studies in the literature have com¬-pared DN versus SWT. Walsh et al. [8] com¬pared the efficacy of SWT versus DN on trigger points in the quadriceps muscle. They performed the interventions only for a week and did not follow their patients. Like the current study, they presented significant pain improvement following both the approaches, but none of them was superior to the other [8]. Rahbar et al. [6] compared the outcomes of DN versus SWT on the pain and function of those resenting from plantar fasciitis in a study on 72 patients. Patients under both treatments developed significant rehabilitation regard¬ing pain and function. The follow-up inves-tigations revealed DN's superiority to SWT within eight weeks after the intervention [6].Further studies assessing DN only have been accompanied by favorable outcomes. Rayegani et al. [7] tried to evaluate the efficacy of DN for the management of trigger points in the trapezius muscle. Their study achieved prom¬ising outcomes in terms of pain relief at rest, at night, and during activity and improvement in the quality of life [7]. Similar results were found in another study with a similar design assessing DN utility on ITBS. The efficacy of DN was to the extent that the patient could lie on the involved side and walk for a more extended period [11]. Another case series revealed outcomes in favor of DN regarding both pain relief and improved function in the short-term as well as the long-term assess¬ment of the patients [12]. Castro-Sánchez et al. made a thorough investigation of DN used for trigger points in latissimus dorsi muscle, multifidus muscle, and quadratus lumborum and reported significant pain relief of all assessed areas [20]. The action mechanism of DN is unknown; however, the mechanical destruction of the dysfunctional endplates responsible for a sustained contraction of muscle fiber due to the continuous release of acetylcholine seems to play the primary role in DN. Therefore, by the destruction of nerve end plates, the muscle fibers contraction terminates, and eventually, the nociceptive impulses to the central nervous system would cease [21, 22].Moghtaderi et al. used SWT for the trigger points in gastrocnemius/soleus muscles. Their investigation was accompanied by consider¬able improvements in the evaluated patients' pain complaints and muscular function [23]. The outcomes were confirmed in another study on cases suffering from trigger points on the quadratus lumborum muscle [24].SWT has been successfully administered for numerous overuse injuries such as patellar tendinopathies, palmar fasciitis, and shoulder calcific tendonitis [25, 26]. SWT's mechanism leading to the promotion of the function and pain relief at the site of these types of chronic injuries is debatable. Increase in the microcir¬culation Improvement in the local microcir¬culation, developing the metabolic activities, and washing the inflammatory agents respon¬sible for pain out from the site of injury are one of the most favored theories about the mechanism of SWT [27,28,29]. This theory was reinforced by the interpretation of the biopsies taken from the site of a trigger point on the lateral femoral epicondyle [30].The hyperstimulation analgesic effect is another mechanism proposed for SWT and is supported due to the intervention's imme¬diate pain relief [31]. We assume that SWT may have an anti-fibrotic effect on the injured soft tissue [32]. This theory is reinforced by the other studies assessing SWT use on the overuse of soft tissue injuries such as adhesive capsulitis or rotator cuff injury [33].The short-term follow-up of the patients was the most prominent limitation of the current study. We have not assessed the post-interven¬tion strength of the muscles, which is strongly recommended for further investigations.

## Conclusion

Based on this study, DN and SWT could remarkably improve pain and function among patients resenting from ITBS; however, our outcomes revealed non-inferiority of each approach over the other. To generalize the outcomes, further studies with a more extended follow-up period are recommended.

## Acknowledgment

The authors of this manuscript want to acknowledge Dr. Ali Safaei for his efforts in conducting and preparing the current study.

## Conflict of Interest

The authors of the current study declare no conflict of interest.

**Table 1:** The Comparison of the Demographic Information Between the Studied Groups.

Variables	Groups	P-value
Dry needling (n=20)	Shockwave (n=20)
Gender, n (%)
Female	15±75	15±75	>0.999^*^
Male	5±25	5±25
Age, mean±SD	49.1±12.31	54.55±16.52	0.24^**^
Body mass index, mean±SD	26.68±3.47	25.25±3.76	0.22^**^
Duration of the ITBS, mean±SD	22±34.58	11.7±19.88	0.11^£^

^*^ Fisher's Exact Test; ^**^ T-test; ^£^ Mann-Whitney

**Table 2:** The Comparison of Pain Severity, LEFS, and ITBL Between the Studied Groups.

Variables	Dry needling (n=20)	Shockwave (n=20)	P-value
Means± SD
Pain
Baseline	8.95±1.14	8.3±1.83	0.38^*^
Immediately by the intervention cessation	3.05±2.08	3.8±2.39	0.39^*^
Within four weeks after the intervention	1.75±1.77	3.6±2.68	0.023^*^
P-value^**^	<0.001	<0.001	
Lower Extremity Function
Baseline	34.9±16.6	38.65±12.52	0.42^δ^
Immediately by the intervention cessation	55.15±17.86	47.9±14.03	0.16^δ^
Within four weeks after the intervention	54.9±17.77	49.7±14.14	0.31^δ^
P-value^£^	<0.001	<0.001	
Length of ITB
Baseline	26.35±5.67	24.4±6.78	0.56
Immediately by the intervention cessation	25.7±6.13	25.2±5.95	0.86
Within four weeks after the intervention	25.9±6.01	25.75±4.71	0.79
P-value^**^	0.93	0.75	

^*^ Mann-Whitney;^ ** ^GEE; ^δ^ Independent t-test; ^£^ Repeated Measure ANOVA
